# Bloodstream Infection among Children Presenting to a General Hospital Outpatient Clinic in Urban Nepal

**DOI:** 10.1371/journal.pone.0047531

**Published:** 2012-10-24

**Authors:** Rahul Pradhan, Umesh Shrestha, Samir C. Gautam, Stephen Thorson, Kabindra Shrestha, Bharat K. Yadav, Dominic F. Kelly, Neelam Adhikari, Andrew J. Pollard, David R. Murdoch

**Affiliations:** 1 Pediatric Research Unit, Pediatric Department, Patan Academy of Health Sciences, Lalitpur, Nepal; 2 General Practice and Emergency Medicine, Patan Academy of Health Sciences, Lalitpur, Nepal; 3 Oxford Vaccine Group, Department of Pediatrics, University of Oxford; and the NIHR Oxford Biomedical Research Centre, Oxford, United Kingdom; 4 Department of Pathology, University of Otago, Christchurch, New Zealand; and Microbiology Unit, Canterbury Health Laboratories, Christchurch, New Zealand; Université Catholique de Louvain, Belgium

## Abstract

**Background:**

There are limited data on the etiology and characteristics of bloodstream infections in children presenting in hospital outpatient settings in South Asia. Previous studies in Nepal have highlighted the importance of murine typhus as a cause of febrile illness in adults and enteric fever as a leading bacterial cause of fever among children admitted to hospital.

**Methods:**

We prospectively studied a total of 1084 febrile children aged between 2 months and 14 years presenting to a general hospital outpatient department in Kathmandu Valley, Nepal, over two study periods (summer and winter). Blood from all patients was tested by conventional culture and by real-time PCR for *Rickettsia typhi*.

**Results:**

Putative etiological agents for fever were identified in 164 (15%) patients. *Salmonella enterica* serovar Typhi (*S.* Typhi) was identified in 107 (10%), *S. enterica* serovar Paratyphi A (*S.* Paratyphi) in 30 (3%), *Streptococcus pneumoniae* in 6 (0.6%), *S. enterica* serovar Typhimurium in 2 (0.2%), *Haemophilus influenzae* type b in 1 (0.1%), and *Escherichia coli* in 1 (0.1%) patient. *S.* Typhi was the most common organism isolated from blood during both summer and winter. Twenty-two (2%) patients were PCR positive for *R. typhi.* No significant demographic, clinical and laboratory features distinguished culture positive enteric fever and murine typhus.

**Conclusions:**

Salmonella infections are the leading cause of bloodstream infection among pediatric outpatients with fever in Kathmandu Valley. Extension of immunization programs against invasive bacterial disease to include the agents of enteric fever and pneumococcus could improve the health of children in Nepal.

## Introduction

Although febrile illness is one of the most common reasons for seeking medical care in Nepal, information on the clinical importance of invasive bacterial infections is often unavailable due to limited diagnostic microbiological facilities. This is especially true of patients who present to hospital outpatient departments and do not need admission, as these patients are rarely investigated.

We have previously shown that vaccine-preventable bacterial infections are important causes of hospitalization in Nepali children [1], [2]. In particular, there is a high burden of enteric (typhoid) fever, which has also been demonstrated in adults [3], [4]. However, the spectrum and burden of invasive bacterial infections among children seen in outpatient clinics remain poorly defined. The hospital outpatient clinic functions as a primary care facility in urban Nepal and it is in this setting that the majority of pediatric febrile illness is managed.

The importance of *Rickettsia typhi* as a cause of febrile illness in South Asia has been increasingly appreciated [3], [4], [5], [6], but there are limited data from pediatric studies. Recently, relatively high rate of murine typhus was reported among febrile adults in Nepal [7], and serological studies performed in various countries outside the region highlight the importance of this disease in children [8], [9], [10], [11].

We describe the etiology and burden of bloodstream infections and murine typhus in febrile children presenting to the pediatric outpatient department of a large district general hospital in Kathmandu Valley, Nepal. In order to address seasonal variations, the study was conducted over two study periods during summer and winter.

## Materials and Methods

### Setting

Nepal is a low income country with an under five year old mortality rate of 50/1000 live births [12]. Kathmandu Valley, the main urban center of Nepal with three districts, has a population of 2.1 million (average population density: 2372/km^2^) of which 29% are under 15 years of age [13]. Situated at an average altitude of 1616 m, the Valley has a sub-tropical cool temperate climate with a hot summer/monsoon season (June to September; average maximum temperature 29°C; rainfall 1200 mm/year) and cool, dry winters (December to February; average minimum temperature 2°C). Overcrowding, insufficient water supply, inadequate sanitation, and pollution are significant problems in Kathmandu Valley. In summer there is a heavy burden of water-borne diseases and in winter respiratory tract infections are predominant. Enteric fever is endemic in the city.

At the time of the study, Patan Hospital was one of only two large hospitals in Kathmandu Valley with a sizeable pediatric department. Patan Hospital accepts patients from all over the Valley. Annually the Pediatric Department cares for over 50,000 outpatients (21% of all hospital outpatient attendances) and accepts approximately 2,700 inpatient admissions. Only 10% of the patients reside outside Kathmandu Valley.

During the study period, pneumococcal conjugate vaccines were not available in the country. Vaccines against *Haemophilus influenzae* type b and typhoid were available within the private market, but they were not widely used.

### Patients

To capture the seasonal spectrum of infectious diseases, the study was conducted during two periods. The summer study took place between April 30 and August 22, 2006, and the winter study between December 15, 2006, and February 27, 2007. Consecutive children between 2 months and 14 years of age presenting to our hospital outpatient clinics with axillary temperature of ≥38°C (recorded in the clinic at the time of presentation) were offered enrollment in the study. Written informed consent was obtained from parents or caregivers of all patients included in the study. Any patient who was hospitalized within 24 hours after their outpatient visit was excluded.

Data were recorded on case report forms and then computerized. Irrespective of severity of disease, blood was drawn from all patients for blood culture, complete blood count and *Rickettsia typhi* PCR. Other tests were performed as indicated by the patient’s clinical condition. The case report forms and laboratory results for all patients were reviewed by one of two senior pediatricians, and a final diagnosis was assigned.

### Laboratory Methods

Blood samples were inoculated into BACTEC™ PEDS PLUS™/F blood culture bottles (Becton Dickinson, USA), incubated at 35°C and processed manually. All bottles were checked twice daily for turbidity and had subcultures performed, irrespective of turbidity, at 12–24 hours and after five days of incubation. Bacterial identification was done by standard microbiological methods and antibiotic susceptibility testing was by disc diffusion using Clinical and Laboratory Standards Institute guidelines [14]. Isolates were sent to Canterbury Health Laboratories, Christchurch, New Zealand, for further confirmation. Complete blood count was performed on the day of the patient’s visit using automatic blood cell counter (PCE-210, Erma Particle Counter). Whole blood samples (stored at −70°C) were tested by real-time PCR for *R. typhi* at Canterbury Health Laboratories using an assay described elsewhere [7].

### Nutritional Status

Weight for age Z-scores were calculated for each subject (EpiInfo 2002). Moderate or severe malnutrition was defined as a Z-score <−2 and severe malnutrition was defined as a Z-score <−3.

### Statistical Analysis

Data from patients with enteric fever were compared with other patients using the chi-square test or Fisher exact test for dichotomous and ordinal variables, and 2-sided Wilcoxon rank sum test and the Student t test for continuous variables. We used multivariable logistic regression analysis to further evaluate variables associated with enteric fever. Enteric fever was the outcome variable in the final model; other variables were those associated with the outcome with *p*<0.1 on bivariable analysis. Likelihood ratio tests were used to compare models with and without interaction terms. Collinearity was tested by a correlation matrix. Data were analyzed using Stata version 11.0 (StataCorp, College Station, TX).

### Ethics Statement

Ethics approval to conduct this study was obtained from Nepal Health Research Council (Ref. no. 686, 46) and Oxford Tropical Research Ethics Committee (OXTREC 026-04, 032-06).

## Results

During the two study periods, 1084 (845 in summer and 239 in winter) febrile children less than 15 years of age were enrolled (Table 1). Of the total, 649 (60%) were male and 227 (21%) had reported taking antibiotics within 48 hours before presentation. Over the entire study period, upper respiratory tract infection and enteric fever were the most common initial clinical diagnoses. Enteric fever was the most common initial diagnosis in summer and respiratory tract infection in the winter.

**Table 1:** Clinical characteristics of febrile children presenting to the Pediatric Outpatient Department at Patan Hospital, Nepal, during the summer and winter study periods.

Characteristics	Summer (n = 845)	Winter (n = 239)
Age (years), median (range)	4 (2 months–14 years)	4 (2 months–14 years)
Male	502 (59%)	147 (62%)
Recruitment rate (no./day)	10	4
Duration of fever (days), median (range)	3 (<1–30)	2 (<1–9)
Temperature at presentation (°C), median (range)	38.3 (38–42)	38.5 (38–40)
Antibiotic use reported within 48 hours before presentation	186 (22%)	41 (17%)
Clinical diagnosis at presentation		
Enteric fever	311 (37%)	23 (10%)
Lower respiratory tract infection	125 (15%)	25 (10%)
Upper respiratory tract infection	216 (26%)	159 (67%)
Acute gastroenteritis/Dysentery	24 (3%)	4 (2%)
Urinary tract infection	28 (3%)	2 (1%)
Viral fever (without obvious focus of infection)	106 (13%)	16 (7%)

### Diagnostic Testing

Whole blood for culture was received from all the enrolled patients and the average blood volume drawn was 2.3 mL. Only 4% had blood volume <1 mL. Only five (0.5%) of the blood cultures yielded an isolate thought to be a contaminant. Whole blood specimens were available from all of the patients for testing by PCR for *R. typhi.*

As presented in Table 2, putative etiological agents for fever were identified in 164 (15%) patients. *Salmonella enterica* serovar Typhi (*S.* Typhi) was the most common organism isolated from blood during both seasons. The median age of patients for bacteremia differed by pathogen (Table 3). The median age for *S*. Typhi, *S.* Paratyphi A and *R. typhi* infection was above five years and for *S. pneumoniae* was below five years.

**Table 2:** Etiological agents identified in febrile children presenting to the Pediatric Outpatient Department at Patan Hospital, Nepal, during the summer and winter study periods.

Bacterial pathogen	Summer (n = 845)	Winter (n = 239)
**Bloodstream isolates (Blood culture)**	131 (16%)	16 (7%)
* Salmonella enterica* serovar Typhi	95 (11%)	12 (5%)
* Salmonella enterica* serovar Paratyphi A	29 (3%)	1 (0.4%)
* Salmonella enterica* serovar Typhimurium	2 (0.2%)	0 (0%)
* Streptococcus pneumoniae*	3 (0.4%)	3 (1%)
* Haemophilus influenzae* type b	1 (0.1%)	0 (0%)
* Escherichia coli*	1 (0.1%)	0 (0%)
**PCR**		
* Rickettsia typhi*	17† (2%)	5 (2%)

† 4 *R. typhi* PCR positive patients had *S*. Typhi and 1 had *S*. Paratyphi A in blood culture.

**Table 3:** Pathogens responsible for blood stream infections according to age group*.

Pathogen†	Isolates	Age Groups (Total patients = 1084)				Median age (IQR)‡
		2 - <6 mo (n = 20)	6 mo - <1 yr (n = 77)	1 - <5 yr (n = 503)	5–14 yr (n = 484)	
**Gram-positive**						
* Streptococcus pneumoniae*	6 (4)	0	0	4 (9)	2 (2)	41 (31–67)
**Gram-negative**						
* Salmonella enterica* serotype Typhi	107 (73)	0	1 (33)	35 (74)	71 (73)	76 (45–118)
* Salmonella enterica* serotype Paratyphi A	30 (20)	0	0	6 (13)	24 (25)	117 (71–139)
* Salmonella enterica* serotype Typhimurium	2 (1)	0	1 (33)	1 (2)	0	16 (12–19)
* Haemophilus influenzae* type b	1 (1)	0	0	1 (2)	0	32
*Escherichia coli*	1 (1)	0	1 (33)	0	0	10
**Total bacterial isolates by blood culture**	147 (100)	0	3	47	97	79 (45–121)
**Rickettsia (PCR)**						
* Rickettsia typhi*	22	1	1	6	14	78 (30–112)
**Total microbiological diagnosis**	169††	1	4	53	111	78 (44–121)

* Data are no. (%) or no. unless stated otherwise. Percents shown for column totals only for bacterial isolates by blood culture.

† Pathogens isolated by blood culture unless indicated otherwise.

‡ Median age in months.

†† 4 patients with *S.* Typhi and 1 patient with *S.* Paratyphi A were PCR positive for *R. typhi.*

Twenty-two (2%) patients had positive *R. typhi* PCR results. Five patients in summer, who were positive for *R. typhi* PCR, also had bacterial isolates in blood culture. Four of these patients had *S.* Typhi and one had *S.* Paratyphi A. There were no dual etiological diagnoses in winter.

### Antimicrobial Susceptibility Testing

Among the 107 isolates of *S.* Typhi, 60 (56%) were resistant to nalidixic acid and, of these, only 3 showed intermediate resistance to ciprofloxacin. Among the 30 *S.* Paratyphi A isolates, 27 (90%) were resistant to nalidixic acid and, of these, 3 showed intermediate resistance to ciprofloxacin. One of the two *S.* Typhimurium isolates showed resistance to nalidixic acid and gentamicin. None of the six *S. pneumoniae* was resistant to penicillin but three were resistant to cotrimoxazole and one showed intermediate resistance to chloramphenicol. The single *H. influenzae* type b isolate was resistant to amoxicillin.

### Clinical Associations

#### Enteric fever

Out of the total 1084 patients, 137 (13%) had culture-confirmed enteric fever. Among 600 total children under five years of age, 42 (7%) had culture-confirmed enteric fever (36 positive for *S.* Typhi and 6 positive for *S.* Paratyphi A). Whereas among 484 total children ≥5 years of age, 95 (20%) had culture-confirmed enteric fever (71 positive for *S.* Typhi and 24 positive for *S.* Paratyphi A).

Compared with all the other patients in the cohort, the patients with culture-confirmed enteric fever were more likely to be older, have fever for more than three days at presentation, present in the summer, be moderately or severely malnourished, and were more likely to have headache, abdominal pain, diarrhea and splenomegaly (Table 4). These children were also less likely to present with cough or rhinorrhea. No significant differences were found between culture positive typhoid and paratyphoid fever cases.

**Table 4:** Demographic, clinical and laboratory features of patients with culture-confirmed enteric fever and their comparison with all the other patients in the cohort*.

Variable	*S.* Typhi/*S.* Paratyphi A bacteremia (n = 137)	Others (n = 947)	*p* value
Demographics			
Age, months, median (range)	81.3 (10.9–167.8)	48.4 (2.6–208)	<0.001
Male sex	74 (54)	575 (61)	0.14
Summer Season	124 (91)	721 (76)	<0.001
Duration of fever, days, median (range)	4 (1–22)	3 (0–30)	<0.001
Duration of fever >3 days	76 (55)	309 (33)	<0.001
Antibiotics taken within 48 hours before presentation	41/125 (33)	186/762 (24)	0.05
Symptoms			
Cough	55/137 (40)	613/946 (65)	<0.001
Shortness of breath	0/137 (0)	7/944 (0.7)	0.31
Nausea/vomiting	49/137 (36)	274/947 (29)	0.10
Diarrhea	21/137 (15)	72/944 (8)	0.003
Abdominal pain	76/131 (58)	311/766 (41)	<0.001
Headache	92/130 (71)	355/710 (50)	<0.001
Joint pain	1/130 (0.8)	10/703 (1)	0.55
Muscle pain	0/130 (0)	23/702 (3)	0.04
Baseline Observations			
Temperature, °C, mean (SD)	38.8 (0.7)	38.3 (1.3)	0.01
Pulse rate (per minute), mean (SD)	121 (15)	124 (19)	0.05
Respiratory rate, breaths per minute, mean (SD)	29 (9)	32 (11)	0.004
Hepatomegaly	16/137 (12)	72/940 (8)	0.11
Splenomegaly	21/137 (15)	77/940 (8)	0.007
Rhinorrhea	28/137 (20)	427/943 (45)	<0.001
Moderate or severe malnutrition	46/110 (42)	242/784 (31)	0.02
Laboratory findings			
Hematocrit, %, mean (SD)	36 (4.5)	35.6 (4.9)	0.38
Leukocyte count, total count/mm^3^, median (IQR)	7250 (6000–9050)	9750 (7100–13600)	<0.001
Leukocyte count >11000/mm^3^	17/136 (13)	368/947 (39)	<0.001

* Data are no. (%) unless stated otherwise. SD, standard deviation; IQR, interquartile range.

On multivariable analysis, culture-confirmed enteric fever was only independently associated with increasing age, diarrhea, duration of fever more than three days, and inversely associated with cough, leukocytosis, and (in winter only) rhinorrhea (Table 5).

**Table 5:** Associations with *S.* Typhi/*S.* Paratyphi A bacteremia on multivariable analysis.

Variable	OR	95% CI	*p* value
Age group<1 year	1.0		
1–5 years	5.49	0.73 to 41.30	0.10
>5 years	10.94	1.46 to 81.85	0.02
Cough	0.48	0.30 to 0.75	0.001
Diarrhea	2.22	1.18 to 4.17	0.01
Duration of fever >3 days	1.64	1.04 to 2.57	0.03
Splenomegaly	1.05	0.55 to 2.01	0.88
Rhinorrheasummer	0.70	0.39 to 1.27	0.24
Winter	0.13	0.03 to 0.64	0.01
WBC >11,000/mm^3^	0.33	0.18 to 0.59	<0.001
Moderate/severe malnutrition	1.36	0.86 to 2.14	0.18

Among blood culture positive patients, those who had leukocyte count <5000/mm^3^ were all positive for *S.* Typhi or *S.* Paratyphi A. Among the culture-confirmed enteric fever patients, fifteen (11%) had leukocyte count ≤5000/mm^3^ and four (3%) patients had a leukocyte count ≥20,000/mm^3^ with ≥50% polymorphonuclear cells; all these four patients were below five years of age.

Nine (2%) of 375 patients who were presumed to have upper respiratory tract infection at presentation, had *S.* Typhi or *S.* Paratyphi A in their blood cultures. Among the patients with duration of fever with rhinorrhea more than five days, 12/46 (26%) had culture-confirmed enteric fever.

#### Murine typhus

There were 22 patients (2%) who were PCR positive for *R. typhi.* Of these, 13 (59%) were male and the median age was 6.4 years. Enteric fever was the most common clinical diagnosis (45%) at presentation for these patients. Compared with all the other patients in the cohort, these patients were more likely to present with nausea or vomiting (50% versus 29.4%; *p* = 0.04) and diarrhea (31.8% versus 8.1%; *p*<0.001), and were less likely to present with cough (36.4% vs. 62.2%, *p* = 0.01). No demographic, clinical and laboratory features distinguished these patients from patients with culture-confirmed enteric fever. None of these patients presented with rash.

#### Pneumonia

Of the 192 (18%) patients with pneumonia as a final diagnosis, only seven (4%) had positive blood cultures (six with *S. pneumoniae* and one with *H. influenzae* type b). All of these seven patients had neutrophil count >11,000/mm^3^ and presented with duration of illness of less than five days.

## Discussion

To our knowledge, this is the first study in the Indian subcontinent to examine bloodstream infections among children up to 14 years of age presenting to an outpatient clinic, the only inclusion criteria for whom was documented fever ≥38°C. In our study, all febrile children were studied rather than a subset of more severely ill children. Every child enrolled had a blood culture performed irrespective of severity of disease. Studies conducted in South Asia that included children either concentrated on pneumonia or acute respiratory infections [15], [16], [17], included more severe patients [18], [19], collected data based on blood cultures received in the laboratory [20], [21], or focused on patients diagnosed with specific disease syndromes such as typhoid fever [22], [23]. Two studies [24], [25] which looked at typhoid and paratyphoid bacteremia are similar to our study; however, they were community-based studies and only the bacteremia rate from patients under five years from one of the two studies can be compared with our results (see below).

Even though we excluded patients severe enough to be hospitalized, some 15% of children with febrile illness who were managed in the outpatient setting in our study had documented bacteremia. This was largely driven by the burden of enteric fever, but we also documented the presence of invasive pneumococcal and *H. influenzae* type b infections. This study also confirms that murine typhus, previously unrecognized as an important pathogen among children in Nepal, is endemic in Kathmandu Valley. We found that distinguishing between enteric fever and murine typhus is clinically difficult.

Enteric fever is an important cause of illness and death in south-central and south-east Asia, particularly among children and adolescents [26]. In this study, *S.* Typhi and *S.* Paratyphi A were the most common bloodstream isolates, comparable to the results found in the Nepal study in adults [4]. Culture-confirmed enteric fever was also relatively common in children aged less than five years, which has been noted in other Asian studies [20], [25], [27]. In children less than five years, *S.* Typhi isolation rate was 6% compared to 4.4% in the study conducted in Bangladesh [24], which had similar inclusion criteria to our study but only for children under five years. However, *S.* Paratyphi A isolation rate in our study was 1% compared to 0.2% in the Bangladesh study. During the last decade, salmonella bacteremia in our hospital patients has more than doubled, and *S.* Paratyphi A as a proportion of all salmonella isolates has risen significantly [28]. The latter has been attributed to the emergence of a single clone of *S.* Paratyphi A in South Asia [29].

In our study, the clinical presentations of *S.* Typhi and *S.* Paratyphi A infections were similar. This is consistent with studies from Indonesia [30] and among adult Nepalis [31] that showed these two infections were clinically indistinguishable and had equal severity. Compared with other patients in the study, children with culture-confirmed enteric fever were more likely to be older, have diarrhea, and have a more prolonged duration of fever, and were less likely to have a raised leukocyte count or cough. While the odds of enteric fever were considerably reduced in children with rhinorrhea during the winter period, one-quarter of all patients with fever and rhinorrhea lasting more than five days were culture-positive for *S*. Typhi or *S.* Paratyphi A. This finding is clinically important as standard practice here has been to avoid blood cultures in outpatients with obvious upper respiratory infections.

Nalidixic acid resistance was more common in *S.* Paratyphi A isolates compared with *S.* Typhi (90% vs 56%). This is in concordance with other studies undertaken in our hospital [31] and in India [32]. Nalidixic acid resistance is a marker of reduced susceptibility to the fluoroquinolone group [33], [34], [35], [36] which may be associated with increased clinical virulence [37] and treatment failure [38], [39], [40]. Widespread use of ciprofloxacin/ofloxacin in Nepal may have driven this increase in fluoroquinolone resistance and alternative approaches to management may be needed, such as azithromycin or gatifloxacin [41].

We did not have any multidrug-resistant salmonella in our study. Nevertheless, there have been reports of extended-spectrum β-lactamase-producing *S.* Typhi in Asia [42], [43] and from travelers returning from Asia [44], [45]. Multidrug-resistant extended-spectrum β-lactamase-producing *S.* Paratyphi A has also been reported from blood isolates in Nepal [46], leading to further concerns about optimal management of the condition where third generation cephalosporins have been widely used for hospitalized cases. Effective typhoid vaccines [47], [48] should be considered for programmatic use in Kathmandu Valley, and paratyphoid vaccines are urgently needed following the emergence of *S.* Paratyphi A as a major cause of enteric fever in the region [28], [46]. Health education, adequate sanitation and the use of chlorinated water will greatly help to reduce the public health burden both now and in the future, but the scale of the problem means that this cannot be resolved rapidly in Kathmandu Valley and immunization is the only intervention which could have a rapid impact.

There is growing interest in the importance of murine typhus in Nepal following the findings of recent studies in adults [3], [7] and given the frequent contact between humans and rodents in urban areas. Rats and other rodents are the natural reservoirs for *R. typhi*, with the rat flea being the transmission vector to humans. Since *R. typhi* antibodies can persist for several months to years following infection [49] and serological detection is non-specific and cross-reactive [4], [50], (and in the absence of convalescent sera), we tested blood samples by real-time PCR assays for *R. typhi* using a recently developed sensitive and specific method [51]. We detected *R. typhi* DNA in the blood from 22 (2%) patients, with a similar prevalence across both seasons. This contrasts with the earlier adult study in Kathmandu Valley that showed an overall prevalence of 7% and winter predominance [7]. This adult study had similar patient inclusion criteria to ours (although hospitalized patients were included) and used real-time PCR for *R. typhi* detection. The other serological study conducted in Nepal [3] among a subset of febrile patients used indirect microimmunofluorescence assay for detection of antibodies to *R. typhi*; this study showed that 26% of their patients were positive for *R. typhi* IgM antibody.

Five of our patients who were positive for *R. typhi* also had positive blood cultures for other bacteria making the significance of the *R. typhi* results uncertain. The collective evidence of recent studies suggests that murine typhus is endemic in Kathmandu Valley, but further focused studies are needed to more precisely determine the burden of disease in children and the clinical significance of a positive PCR in suitable controls. This is especially important given its clinical similarity with enteric fever as this pediatric study and the adult study [7] emphasize the similar clinical features of blood culture positive enteric fever and murine typhus. The absence of rash has been consistently reported in murine typhus from Nepal [7]. Murine typhus should be considered as an alternative diagnosis in patients suspected with enteric fever in Nepal, especially in those not responding to first-line antimicrobial drug therapy to enteric fever [7].

This study has several limitations. For practical reasons we could only conduct the study during two brief periods to capture the seasonal variation of infectious diseases in the Valley. Consequently, we lack data over a complete year or over multiple years to account for annual variation in disease rates. We did not follow-up patients for the study purpose and, therefore, have no data on outcomes once they were sent home from the clinic. We did not test for HIV infection given the low seroprevalence among children admitted with febrile illness in Nepal. Of 485 serum samples tested from admitted febrile children around the same time, only one (0.2%) was positive for HIV antibodies [1]. Other possible pathogens detected by serological diagnoses such as *Leptospira* spp., *Orientia tsutsugamushi*, dengue viruses, as shown in the adult population [3], [4], were not included in this study because of limited resources and blood volumes.

However, this study has highlighted the burden of enteric fever among children in Kathmandu Valley, including children considered well enough to be managed in an outpatient/primary care setting. Sentinel studies like this in resource poor settings can define the burden, pattern and treatment of infectious diseases and alter healthcare delivery. However, since the spectrum of pathogens, incidence of diseases and antimicrobial susceptibility change over time, the data should be monitored continuously to allow an appropriate clinical response and healthcare planning. The data show that immunization programmes against invasive bacterial disease could improve the health of children in Kathmandu Valley and that there is a pressing need for effective vaccines against the agents of enteric fever.
